# Chemoinformatic studies on some inhibitors of dopamine transporter and the receptor targeting schizophrenia for developing novel antipsychotic agents

**DOI:** 10.1016/j.heliyon.2020.e04464

**Published:** 2020-07-28

**Authors:** Sabitu Babatunde Olasupo, Adamu Uzairu, Gideon Adamu Shallangwa, Sani Uba

**Affiliations:** a National Agency for Food and Drug Administration and Control (NAFDAC), Nigeria; b Department of Chemistry, Ahmadu Bello University Zaria, Nigeria

**Keywords:** Pharmaceutical chemistry, Physical chemistry, Theoretical chemistry, Qsar, Disorder, Antipsychotic, OECD, Drug, Depression

## Abstract

Chemoinformatic studies were carried on some inhibitors of dopamine transporter to develop a predictive and robust QSAR model and also to elucidate binding mode and molecular interactions between the ligands (inhibitors) and the receptor targeting schizophrenia as novel Antipsychotic agents. Density Functional Theory (DFT) approach was utilized to optimize the ligands at B3LYP/6-31G∗ at the ground state and Multi-linear regression of the genetic function approximation (MLR-GFA) method was employed in building Penta-parametric linear equation models. The best model with statistically significant parameters has squared correlation coefficient R^2^= 0.802, adjusted squared correlation coefficient R^2^_adj_ = 0.767, Leave one out (LOO) cross-validation coefficient (Q^2^) = 0.693, lack of fit score (LOF) = 0.406, R^2^_Test_ = 0.77, *Y*-randomization test (cR^2^p) = 0.714, Chi-squared (χ^2^) =0.026, bootstrapping (Systematic errors = 0.272) and Variance Inflation Factor (VIF) <2 . The obtained results were compared with standard validation parameters to ascertain the predictivity, reliability, and robustness of the model. Also, the mechanistic interpretation of the descriptors found in the model revealed that two out of five descriptors; MATS7s (32.3%) and RDF95m (30.4%) having pronounced influence on the observed antipsychotic property of the compounds evidenced by their highest percentage contributions. More so, the molecular docking investigation showed that the binding affinity of the selected ligands ranges from -10.05 to -9.0 kcal/mol and with ligand 21 possessed the highest binding affinity (-10.05 kcal/mol). Furthermore, all the selected ligands displayed hydrogen bonds and hydrophobic interactions with the amino acid residues of the target (4M48) which could account for their higher binding energy. Our findings revealed that the developed model passed the general requirements for an acceptable QSAR model and also satisfied the OECD principles for model development. Hence, the developed model would be practically useful as a blueprint in developing novel antipsychotic agents with improved activity for the treatment of schizophrenia mental disorder.

## 1 Introduction

Schizophrenia is a psychiatric disorder that frequently involves a composite genetic tendency as well as susceptibility to certain environmental factors [1, 2]. It is a chronic and debilitating mental disorder characterized by disordered thoughts, abnormal behaviors, and anti-social behaviors, meaning that the person with schizophrenia problem does not identify with reality at times [3]. Meanwhile, Psychosis refers to a state in which an individual experiences a false sensation and this includes auditory, visual, and tactile sensations of things that are not real, and feelings that something strange is going on [4, 5]. Symptoms of Schizophrenia and Psychosis are related and they include; hallucinations, delusions, incoherent speech, dangerous behavior, unusual movements, problems at public places and detached manner with the inability to express emotion, apathy and lack of enthusiasm [6, 7]. Persistent in symptoms of Psychosis may be a risk that the affected individual could be experiencing manifestations of schizophrenia or mental disorders that are considered as the precursors of schizophrenia [6]. Antipsychotic drugs (APDs) are the backbone in the treatment of schizophrenia. The available APDs exhibit a broad range of mechanisms and act on receptors of diverse biogenic monoamine neurotransmitters [8].

Dopamine is the main neurotransmitter involved in the pathophysiology and treatment of schizophrenia [9]. Dopamine pathways have been well interpreted by Positron emission tomography (PET) with different radiotracers and these PET tracers have been used to elucidate various aspects of divergent dopaminergic transmission in schizophrenia [10, 11, 12]. Dopamine (DA) receptor and transporter dysfunctions play an important role in the pathophysiology of neuropsychiatric mental disorder including anxiety disorder (AD), major depressive disorder (MDD), bipolar disorder or depressive state and schizophrenia disease [13]. All effective antipsychotic medications achieve their efficacy by targeting the dopaminergic system, sub-chronic blockage of 60–80% of dopamine D_2_ receptors is considered to underlie treatment efficacy in schizophrenia [14]. Clozapine was reported to be the most effective pharmacotherapy for the treatment of schizophrenia since the introduction of conventional antipsychotic drugs in 1950s [15, 16]. Despite its superior efficacy and potential to reduce substantially the morbidity of schizophrenia and improve the outcomes of patients, Clozapine has not been used on a widespread basis due to its potential for agranulocytosis [15]. Even though other antipsychotic drugs such as Cariprazine, risperidone, etc. were discovered after Clozapine, none of the beneficial effects for any of the medication strategies were considered to be of clinically significant magnitude [15, 17, 18]. More worrisome is the incidence of “ultra-resistance” cases where patients with schizophrenia respond neither Clozapine nor any other antipsychotic drugs [7]. Since existing antipsychotic drugs for the treatment of schizophrenia are faced with challenges of adverse side effects, patients' ultra-resistance, and insignificant clinical benefits, the need for a continuous search for potent and less toxic antipsychotic drugs has become very necessary. Although, drug discovery and development are very tedious scientific exercises owing to the stupendous time factors and resources involved, thus the application of chemoinformatic study to overcome these bottlenecks is very important at this time. Because of these, some of the inhibitors (ligands) of Dopamine Transporters and the receptor (Dopamine transporter elucidates antidepressant mechanism) targeting schizophrenia that is well documented in the CHEMBL Database [19] and the Protein Data Bank respectively were studied as to harness the structural features influencing the observed antipsychotic activity of the inhibitors as well as to elucidate the binding mode and molecular interactions between the ligands and the receptor targeting schizophrenia. In consequence, the results of the study are hoped to provide necessary information for the identification and development of novel Antipsychotic agents with improved activity for the treatment of schizophrenia and other related mental disorders.

## 2 Computational methods

### 2.1 Experimental data set

A data set of 44 inhibitors of DAT was collected from the CHEMBL Database [19]. The general molecular structures of the compound's vis-a-vis their CHEMBL number are contained in Supplementary Table SD1. The anti-psychotic activities expressed in nM were converted into the corresponding pKi (pKi = -log_10_ pKi) values which are used as dependent variables in this study.

### 2.2 Molecular optimization and descriptors calculation

2D structures of the compounds were drawn using Chemdraw software and the spatial conformations of the compounds were determined using the Spartan 14 V1.1.4 wavefunction software package [20]. The molecular structures were minimized by Molecular Mechanics Force Field (MMFF) calculations to remove strain energy before subjecting it to complete geometry optimization with the aid of Density Functional Theory (DFT) at B_3_LYP/6-31G∗ basis set. The molecular descriptors were calculated using the PaDel descriptor tool kit, Spartan 14 software and ChmBio3D Pro 12.0.1V software [21]. More than a thousand descriptors comprising of 0D, 1D, 2D, and 3D types were generated for each molecule. The descriptors were correlated with the antipsychotic activity of the molecules using Pearson's correlation matrix to select the suitable descriptors for Genetic Function Approximation (GFA) analysis based on the correlation coefficients.

### 2.3 Experimental data set division

The data set (44 compounds) were split into a training set of 34 compounds (77%) which was used to develop the models and test set that is made up of 10 compounds (23%) that was utilized externally to validate the predictivity of the models [22, 23] by employing Kennard stone algorithm method with the use of Dataset Division GUI 1.2” software [24, 25] in line with the optimum splitting pattern of data set in QSAR study [26].

### 2.4 Model building and evaluation of chemometric parameters

Different possible combinations of descriptors were subjected to Genetic Function Approximation (GFA) with the experimentally determined biological activity on a logarithmic scale (pKi) as the dependent variable and the descriptors as the independent variables. Out of the three generated GFA models, the best (Model-1a) which is statistically significant and with the smallest LOF score was selected. The use of Friedman's lack-of-fit (LOF) measure has several advantages over the regular least square error measure in evaluating the quality and fitness of a model [23]. Mathematically, the Friedman lack-of-fit (LOF) is expressed [27, 28] by Equation (1) below as;

(1) $$LOF=\frac{SSE}{{\left(1-\frac{C-dp}{M}\right)}^{2}}$$

SSE = sum of squares of errors, c = number of terms in the model, other than the constant term, d = user-defined smoothing parameter, p = total number of descriptors contained in all model terms while M represents the number of samples in the training set [29].

### 2.5 QSAR model validation and principles of OECD

Validation is a decisive step in a QSAR modeling in which the predictivity, reliability, and significance of the procedures are confirmed in developing a model [30]. The principles of Organization for Economic Co-operation and Development (OECD) that constitute a conceptual framework for validating a QSAR model were employed in validating the model, that is, a well-defined End-point measured must exist, a univocal algorithm must be used, a defined applicability domain, appropriate statistical evaluation of the models must be carried out (i.e. internal and external validations using the training set and the test set respectively) and mechanistic interpretation of the models must be established [31, 32].

### 2.6 Model validations and procedures

A quest to develop a globally acceptable QSAR model and to ensure compliance to OECD Principles of model validations, appropriate statistical evaluation and validation of the models were investigated using both internal and external validations procedures.

#### 2.6.1 Internal and external validation methods

Internal validation is assessed using the data that created the model via the methods of least squares fit (*R*^2^), cross-validation coefficient (*Q*^2^), adjusted *R*^2^ (*R*^2^_adj_), the difference between R^2^ and Q^2^ (R^2^ - Q^2^), Chi-squared (χ^2^) and Root-mean squared error (RMSE). The values of these parameters were compared with the minimum criterion for robust QSAR models in Table 1 [30]. The R^2^ value is interpreted as the proportion of variation in a dependent variable that is explained by the model. R^2^ is expressed by this formula:

(2) $${\text{R}}^{2}=\frac{SSR}{SST}=\frac{SST-SSE}{SSE}$$

Where SST = total sum of squares, SSR = regression sum of squares, and SSE = minimum sum of squared residuals of any linear model. R^2^ value varies directly with the increase in some descriptors, thus, R^2^ cannot be a useful measure for the goodness of model fit. Therefore, R^2^ is adjusted for the number of explanatory variables in the model [30, 33]. The adjusted R^2^ is defined as

(3) $${\text{R}}_{\left(\text{adj}\right)}^{2}=1–\left(1-{\text{R}}^{2}\right)\frac{n-1}{n-p-1}=\frac{\left(n-1\right)R^{2}-1}{n-p+1}$$

p = number of descriptors in the model.

**Table 1:** Computed chemometric validation parameters of the model.

S/N	Chemometric validation parameters	Description	Threshold value	Calculated value	Comments
1.	R^2^	Co-efﬁcient of determination for internal validation (Training set)	≥0.6	0.802	goodness-of-fit
2.	R^2^_Ext_	Co-efﬁcient of determination for external validation (test set)	≥0.5	0.773	good predictivity
3.	R^2^_adj_	Adjusted R-squared	>0.6	0.767	goodness-of-fit
4.	Q^2^_cv_	Cross-Validation Co-efﬁcient	>0.5	0.693	Passed and model is acceptable
5.	R^2^-Q^2^_cv_	Difference between R2 and Q	≤0.3	0.109	Passed and model is acceptable
6.	Friedman LOF	Friedman LOF	-	0.406	statistically significance
7.	χ^2^	Chi-squared	<0.5	0.026	good predictivity
8.	RMSE	Root-mean squared error	≤1.0	0.448	good predictivity
9.	_c_R^2^p	Coefficient of determination for *Y*-randomization	>0.5	0.714	Robustness
10.	N_B_	No. of Bootstrap Models	-	10,000	Good internal predictivity
11.	Biasˆ2	Bias or systematic errors	-	0.272	Good internal predictivity
12.	MSE	Mean square error	-	0.251	Good internal predictivity
13.	RMSEP	Root-mean squared error of prediction	-	0.50112	Good internal predictivity
14.	Variance	Variance or errors introduced by models	-	0.055	Good internal predictivity
15.	DF (5, 28)	Degree of Freedom	>2.09	33	statistically significance
16.	|r0ˆ2-r'0ˆ2|		<0.3	0.0512	Passed and model is acceptable
17.	k	slopes k (predicted against experimental activity) of the regression lines through the origin	0.85 < k < 1.15	1.022	Passed and model is acceptable
18.	k'	slopes k’ (experimental against predicted activity) of the regression lines through the origin	0.85 < k'<1.15	0.976	Passed and model is acceptable
19.	[(rˆ2-r'0ˆ2)/rˆ2]		<0.1	0.067	Passed and model is acceptable
20.	r^2^_m_	external predictability of the selected model	≥0.5 (or close to 0.5)	0.689	good external prediction
21.	[(rˆ2-r0ˆ2)/rˆ2]		<0.1	0.00067	statistically significance
22.	VIF	Variance Inflation Factor	<10	<2	orthogonal and statistical significance.
23.	t-test	t-Statistic value	>2	>10	statistically significance

The LOO cross-validated coefficient (Q^2^) is given by;

(4) $${\text{Q}}^{2}=\;1-\frac{\sum {\left(A-B\right)}^{2}}{\sum {\left(B-C\right)}^{2}}$$

where A and B represent the predicted and observed activity respectively of the training set and C = mean activity value of the training set.

The Chi-squared (χ^2^) and Root-mean squared error (RMSE) for validating the models and error checking to determine if the model possesses the predictive quality reflected in the R^2^ are expressed by Eqs. (5) and (6) respectively as;

(5) $$X^{2}=\sum_{i=1}^{n}\left(\frac{{\left(y_{i}-\hat{y_{i}}\right)}^{2}}{\hat{y_{i}}}\right)$$

(6) $$RMSE=\;\sqrt{\left(\sum_{i=1}^{n}\;\frac{{\left({\hat{y}}_{i}-\;y_{m}\right)}^{2}}{n-i}\;\right)}$$

y and ŷ represent the experimental and predicted activity for each compound in the training set, ym denotes the mean of the experimental activities, and n is the number of molecules in the study compounds.

Since the real predictive ability of a QSAR model cannot be judged or guaranteed solely base on internal validation [29], so the only way to establish the true predictivity of a model is to compare the predicted and observed activities of the external test set of compounds that were not employed in the model building [30], hence, external validation using test set data becomes a sine qua non to ascertain quality assurance and predictive power of the model. Therefore, the predicted R^2^_ext_ (external validation) of the model is computed by using the Equation (7) below.

(7) $${\text{R}}_{\text{ext}}^{2}=\;1\;-\frac{\sum {\left[w-T\right]}^{2}}{\sum {\left(T-X\right)}^{2}}$$

W and T symbolize predicted and observed activity values respectively of the test set compounds and X indicates the mean activity value of the training set.

#### 2.6.2 Prediction analysis

Bias-Variance Estimator software that uses bootstrapping as a resampling technique obtained from DTC lab website was engaged to examine the prediction error analysis which depends on bias-variance estimation evaluate the role of both the prediction errors, that is, systematic error (bias) and random error (variance) in the developed model [34]. The parameters bias^2^, variance and mean square error (MSE) were computed by using equations 8a-d below;

8a $$Bias^{2}=\;\frac{1}{nc}\sum_{i=1}^{nc}{\left(\bar{y}{\;}_{pred(i)}\;-y_{exp(i)}\right)}^{2}\;$$

8b $${\bar{Y}}_{Pred\left(i\right)}^{B}=\;\frac{\sum_{j=i}^{nB}\;\;y_{Pred\left(i\right)}^{B\left(j\right)}}{n_{B}}$$

8c $$Variance=\;\frac{1}{nc}\sum_{i=1}^{nc}\frac{1}{nB}\sum_{j=1}^{nc}\;{\left(y_{Pred(i)}^{B(j)}-\;{\hat{y}}_{Pred\left(i\right)}^{B}\right)}^{2}\;$$

8d $$MSE=\sum_{i=1}^{nc}\frac{{\left(y_{\exp(i)}-\;y_{pred(i)}\right)}^{2}}{nc}\;$$

Where n_c_ = number of compounds in the test set, y_exp(i)_ = experimental response value of the compound ‘i’, ${\hat{y}}_{Pred\left(i\right)}^{B}$= mean predicted response value of compound ‘i’ from ‘n_B_’ bootstrap models, $y_{Pred\left(i\right)}^{B\left(j\right)}$ = predicted response value of compound i from the bootstrap model ‘j’, n_B_ = number of bootstrapping models produced, $y_{pred\;\left(i\right)}$ = predicted response of compound i from the model.

#### 2.6.3 Evaluating applicability domain of the model

Assessing the applicability region of a model is a crucial procedure in establishing the ability of the developed model weather it can make a reliable prediction within the chemical region or otherwise for which the model was built [23, 35]. For evaluating the applicability domain of the model, both the leverage approach and Euclidean Based Applicability Domain methods were adopted in this study.

The leverage of a given data set of compounds hi, can be defined mathematically as;

(9) $$H_{i}=x_{i}{\left(X^{T}X\right)}^{-1}X_{i}^{T}$$

where xi the descriptor row is the vector of the considered compound *i*, hi is the n x k descriptor matrix of the training set compound used to generate the model.

The warning leverage (*h∗)* is the limit of normal values of x outliers which can be estimated by the Equation (10) below;

(10) $$h^{*}=\frac{3\left(p+1\right)}{n}$$

where n is the number of training compounds and P represents the number of predictor variables in the model.

**Figure 1:**
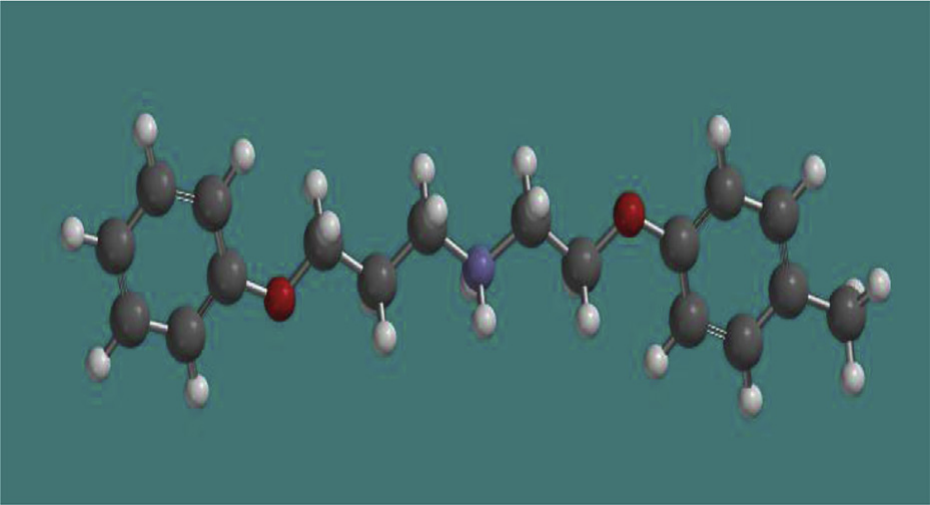
3D chemical structure of ligand 30 with the highest experimental activity (pKi = 9.51).

A developed model is adjudged to be reliably predicted if the leverage hi < h∗ for the investigated compounds. The significance area of the model in terms of chemical space is visualized by Williams's plot (Figure 4: plot of standardized residuals versus leverage values) (see Figure 1).

While the Euclidian distance is expressed mathematically as;

11 $$EDij={\left[\Sigma \text{wk}{\left(x_{k}^{i}-x_{k}^{j}\right)}^{2}\right]}^{1/2}$$

The difference, $x_{k}^{i}$− $x_{k}^{j}$ represents the distance of the test set compounds from the training set compounds and wk is a weighted vector corresponding to the importance of the *k*^th^ descriptor in the model calculated using auto-scaled descriptors, $x_{k}^{i}$ and $x_{k}^{j}$ represent compounds from the test set and training set respectively [36]. The weighting makes it possible to account for the relative contribution of each variable to the similarity and improves the detection of the AD of the model [37]. Computed chemometric validation parameters [30, 38, 39] of the developed model are reported accordingly.

**Model 1**:

12 pKi = 2.534084591a +14.767585116b + 3.144595958c - 13.880313054d - 4.924640371e+ 6.463616422

n = 34, Friedman LOF = 1.072, R^2^ = 0.802, R^2^adj. = 0.767, Q^2^ = 0.693, F-value = 22.691, Min. expt. error for non-significant LOF (95) = 0.406.

Using the formula in Equation (7), the predicted R^2^ value for the test set is calculated as follows;

$$R_{ext}^{2}=1-\frac{\sum {\left[w-T\right]}^{2}}{\sum {\left(T-X\right)}^{2}}=1-\frac{2.511}{10.804}=0.770$$

#### 2.6.4 Molecular docking analysis

Molecular docking analysis was investigated on the studied compounds to elucidate the molecular interactions between the target (receptor) and the inhibitors (ligands) and also to visualize binding interactions as well as to identify the inhibitors with the best binding affinity to the receptor. The X-ray structure of dopamine transporter elucidates the antidepressant mechanism (receptor) obtained from Protein Data Bank (www.rcsb.org) with PDB code 4M48 [40] was used for this investigation. 2D structure of the ligands (Dopamine Transporter Inhibitors) were optimized and saved as PDB files (prepared ligands) using Spartan 14V 1.1.4 software [23]. Also, the receptor was prepared by importing the 3D crystal structure of the receptor (Dopamine transporter elucidates antidepressant mechanism) downloaded from Protein Data Bank into Discovery Studio Visualizer for the addition of Hydrogen and removal of a water molecule, heteroatoms and co-ligands from the raw receptor and consequently saved as PDB file (prepared receptor).

The molecular docking analysis was successfully carried out by using AutoDock Vina version 4.0 of Pyrex software [23]. The results of docking investigations were computed, analyzed and visualized via Discovery Studio Visualizer software.

## 3 Results and discussion

### 3.1 Chemoinformatic investigation and validations of the model

**Figure 2:**
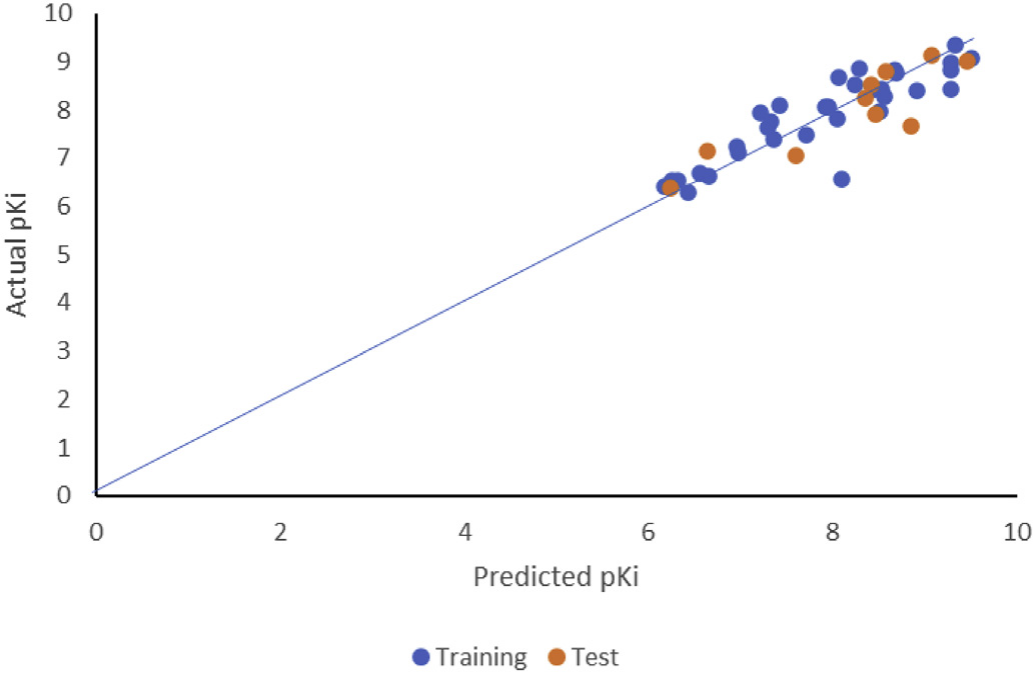
A plot of experimental pKi against predicted pKi (Training set & Test set).

**Figure 3:**
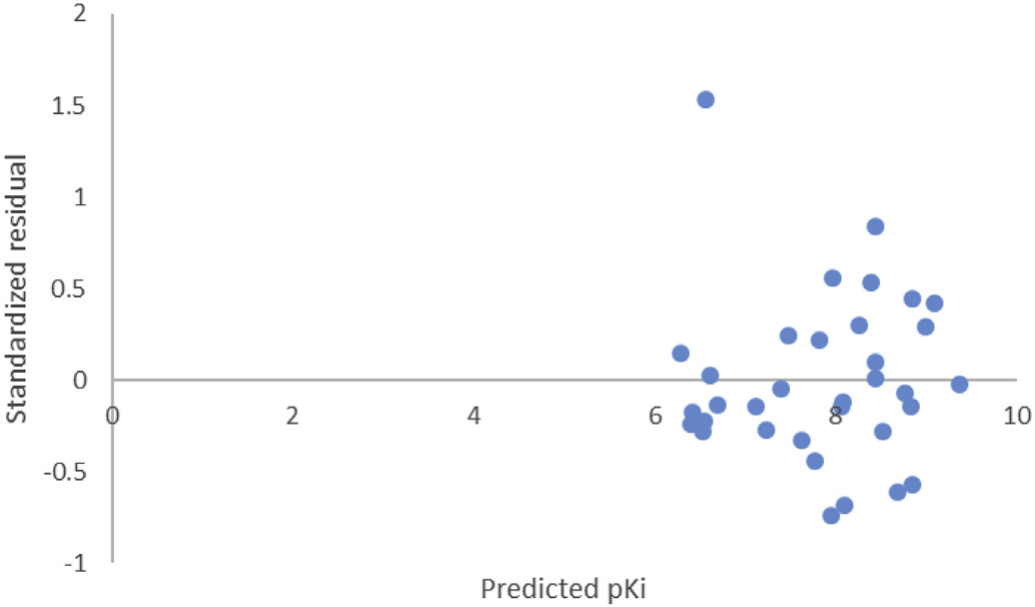
A plot of standardized residual against predicted pKi of the compounds (Training set).

The best model (Model 1**)** was selected as the optimal model because of its statistically significant output in predicting the antipsychotic activity of the studied compounds vis-a-vis the validation parameters. The details of the descriptors that appeared in the model are presented in Table 3. The optimal model proves to be in excellent agreement with the threshold for a generally acceptable model as reported in Table 1 (R^2^ = 0.802, R^2^_adj_ = 0.767, Q^2^ = 0.693, R^2^_ext_= 0.77). This suggests that the optimal model is very predictive and reliable [24, 30]. Also, the plot of predicted pKi against observed pKi as depicted in Figure 2 shows that the model is well trained and correctly predicted the antipsychotic activity of the compounds, an indication of goodness and stability of a model [38]. More so, the plot of observed pKi versus residual pKi (Figure 3) reveals that there was no systemic error in the model developed as the propagation of residuals was observed on both sides of zero [23] [41]. To estimate the extent of prediction errors, the bootstrapping resampling technique was employed in developing 10000 bootstrap samples [34]. The goodness of the prediction of the model was further established by the low estimated values of bias, variance and mean square errors as reported in Table 1. Error checking by applying Chi-squared (χ^2^) and Root-mean squared error (RMSE) procedures were also evaluated as to prove the predictive quality of the model reflected in the R^2^. The chi-squared (0.026) and RMSE (0.448) values obtained (Table1) are indications of a good predictivity of the model [30]. Also, to ascertain the quality assurance of the bioinformatics parameters in the model, Prediction Reliability Indicator 1.0 software was utilized [42]. The model displayed good prediction quality for seven of the test compounds with composite score 3 and moderate prediction for the remaining 3 compounds with composite score 2, more so, all the test compounds were fell within the region of Applicability domain while the values for the data set were in closeness with the mean value of the training set molecules (Table 2). Furthermore, to determine the possibility of multicollinearity between the descriptors used in the model, the variance inflation factors (VIF) of all the descriptors in the model were computed (Supplementary Table SD5) using Equation (13). The corresponding VIF values of the five descriptors used in the optimal model (Model 1) were less than the critical value of 10, a good indication that the developed model is statistically significant, and the descriptors were found to be reasonably orthogonal [23, 43].

(13) $$VIF=\frac{1}{1-R^{2}}$$

where R^2^ is the correlation coefficient of the multiple regression between the variables within the model.

**Table 2:** Prediction Reliability Indicator parameters of the model.

Test Comp No	Score (MAE-LOO)	Score (Proximity Train YObs Mean)	Score (Similarity based AD)	Composite Score	Prediction Quality	Yobs (Test)	YPred (Test)	Abs Prediction Error	Score (Abs Prediction Error)	AD status	Closest Training Compound
1	2	3	3	3	Good	7.602	7.067	0.535	2	In	2
17	1	3	3	2	Moderate	8.585	8.792	0.207	3	In	40
19	2	3	3	3	Good	8.42	8.536	0.116	3	In	18
23	2	3	3	3	Good	6.64	7.148	0.508	2	In	24
26	2	3	3	3	Good	6.24	6.398	0.158	3	In	24
34	2	3	3	3	Good	9.08	9.134	0.054	3	In	38
35	2	3	3	3	Good	9.46	9.014	0.446	3	In	30
37	2	3	3	3	Good	8.36	8.264	0.096	3	In	36
42	1	3	3	2	Moderate	8.469	7.911	0.558	2	In	5
43	1	3	3	2	Moderate	8.854	7.686	1.168	1	In	29

More so, the inhibitory activity (pKi) of the experimental, predicted and residual values of the studied compounds were reported in Supplementary Table SD1. Lower residual value (differences between experimental and predicted activity) obtained also substantiate the good predictivity of the model. Likewise, the Y-Randomization test was investigated to examine the robustness and determine the stability of the model. The results presented in Supplementary Table SD4 showed a low R^2^ and Q^2^ values which are in agreement with chemometric validation parameters reported in Table 1. This is an excellent indication that the derived model is very robust, good and dynamic while its cRpˆ^2^ value of 0.7137, a value greater than 0.5 further proved that the model is not only inferred by chance but also very predictive [24, 44].

**Figure 4:**
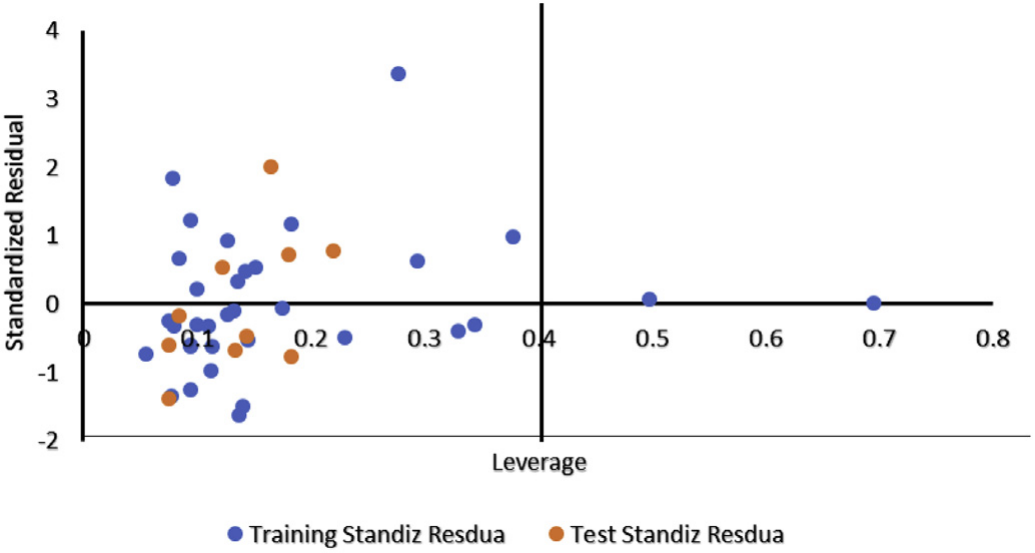
A William's plot of standardized residual against leverage.

**Figure 5:**
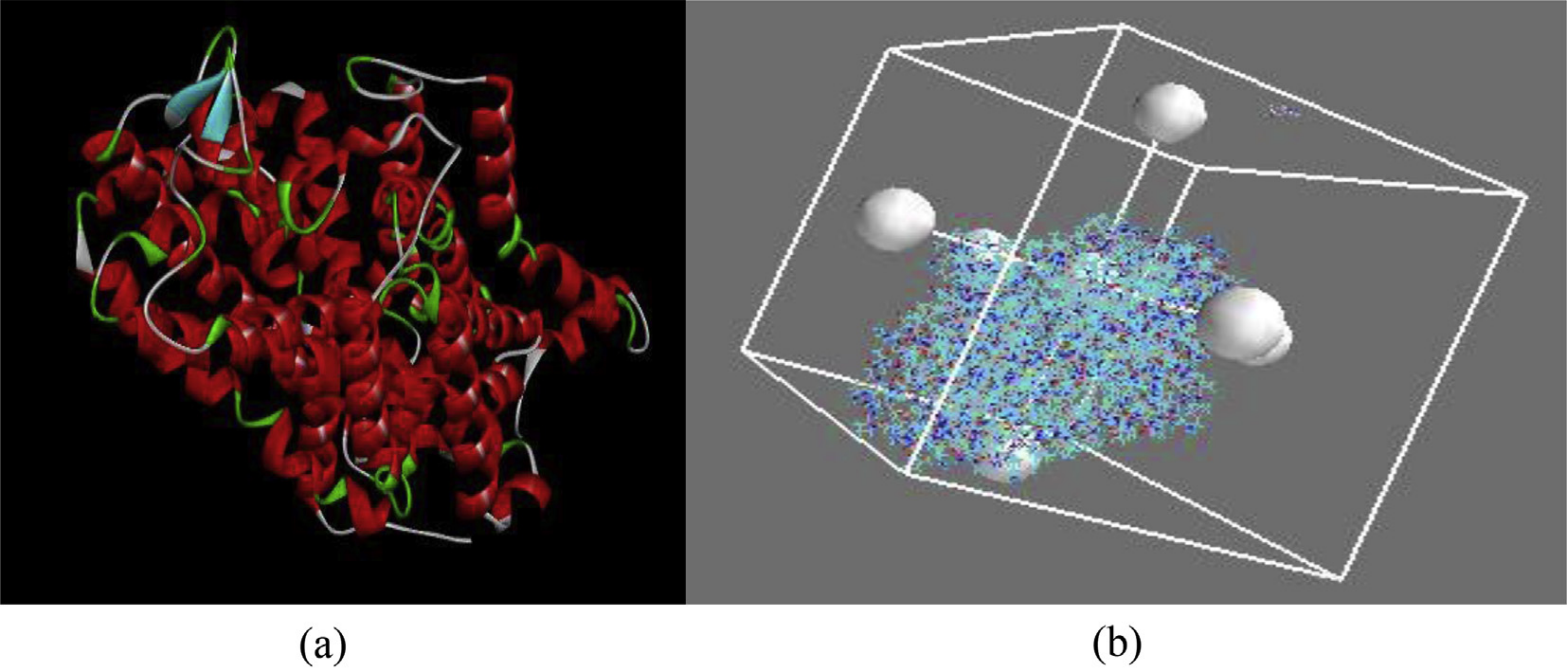
(a) 3D structure of the prepared receptor (b) Docking grid of ligand 21.

The applicability domain of the optimal model (model 1) was equally investigated for the training set and test set using both Euclidean based and Leverage approach procedures. The results (Supplementary Table SD3 and Figure 4) revealed that all the compounds of the test set fell within the applicability domain of the model. However, compounds 44 and 9 of the training set were identified as possible outliers in the two procedures employed as their normalized mean distance scores as well as their leverage values fell outside the domain (Supplementary Table SD2 and Figure 4) which could be considered as structural outliers (influential compounds) (see Figure 5).

### 3.2 Mechanistic interpretation and elucidation of descriptors in the model

The bioinformatics analysis result revealed that the antipsychotic activity of the studied compounds is influenced by the descriptors; ATS7m, MATS7s,VR2_Dzp, RDF95m and RDF150 denoted as a,b,c,d and e respectively in Table 3. Since pKi = -log_10_[pKi], the positive coefficients of the descriptors; a, b, and c imply that the pKi of the compounds against DAT decreases with an increase in values of these descriptors. Thus, to enhance the antipsychotic activity of these compounds, the values of these descriptors should be appreciably high since the activity is inversely proportional to the concentration. Conversely, the descriptors; d and e have negative coefficients as depicted in the model (Equation (12)) (see Table 4). It suggests that the inhibitory activity (pKi) of the ligands decreases with a decrease in the values of these descriptors. Hence, to enhance the inhibitory potency of DAT, the values of these descriptors should be significantly low. More so, Pearson's correlation matrix was employed to examine the inter-correlation among the descriptors of the model and also the percentage contribution of the descriptors to the observed antipsychotic activities of the compounds was also determined (Table 5). The fact that the obtained results for Pearson correlation coefficients for each pair of descriptors were less than 0.5, is a clear indication of insignificant inter-correlation among the descriptors in the model [23]. The descriptors MATS7s and RDF95m displayed a predominant effect (32% and 30.4% respectively) on the observed antipsychotic activity of the compounds. Descriptor **MATS7s** is a descriptor of molecular volume and is defined as Moran autocorrelation weighted by van der Waals volumes. Van der Waal volume is the volume occupied by a molecule or individual atoms of a molecule. Its positive correlation with pKi in the model implies that the higher the descriptor value in a molecule, the lesser the pKi of the molecule and the better its inhibitory activity against the target protein (receptor). Likewise, the descriptor **RDF95m** is a descriptor of molecular mass and is also defined as a Radial distribution function weighted by relative mass. The negative correlation of the descriptor with pKi in the optimal model shows that the lower its value in a molecule, the lesser the pKi of the molecule and the better the inhibitory activity against the receptor.

**Table 3:** Symbols and definition of the descriptors found in the model.

S/N	Descriptor Notation	Descriptor Symbol	Definition
1	A	ATS7m	autocorrelation descriptor, weighted by scaled atomic mass
2	B	MATS7s	Moran autocorrelation - lag 7/weighted by van der Waals volumes
3	c	VR2_Dzp	Normalized Randic-like eigenvector-based index from Barysz matrix/weighted by polarizabilities
4	d	RDF95m	Radial distribution function - 095/weighted by relative mass
5	e	RDF150p	Radial distribution function - 150/weighted by relative polarizabilities

**Table 4:** External validation table for model 1.

S/N	T	W	X	(W-T)2	(T-X)2
1	7.602	7.067	7.84	0.286	0.057
2	8.585	8.792	7.84	0.0429	0.555
3	8.42	8.536	7.84	0.013	0.336
4	6.64	7.148	7.84	0.258	1.44
5	6.24	6.398	7.84	0.025	2.56
6	9.08	9.134	7.84	0.003	1.538
7	9.46	9.014	7.84	0.199	2.624
8	8.36	8.264	7.84	0.009	0.270
9	8.469	7.911	7.84	0.311	0.396
10	8.854	7.686	7.84	1.363	1.028
				∑=2.511	∑=10.804

**Table 5:** Pearson's correlation matrix for descriptors in Model 1.

	*pKi*	*a*	*B*	*c*	*d*	*E*	% contribution
pKi	1						
A	0.007555	1					5.5
B	0.432019	-0.22716	1				32.3
C	0.229263	0.354901	-0.03857	1			6.9
D	-0.32413	0.462982	0.094501	0.163562	1		30.4
e	-0.40256	0.265401	0.180475	0.071903	0.138036	1	10.8

### 3.3 Result of molecular docking analysis

**Figure 6:**
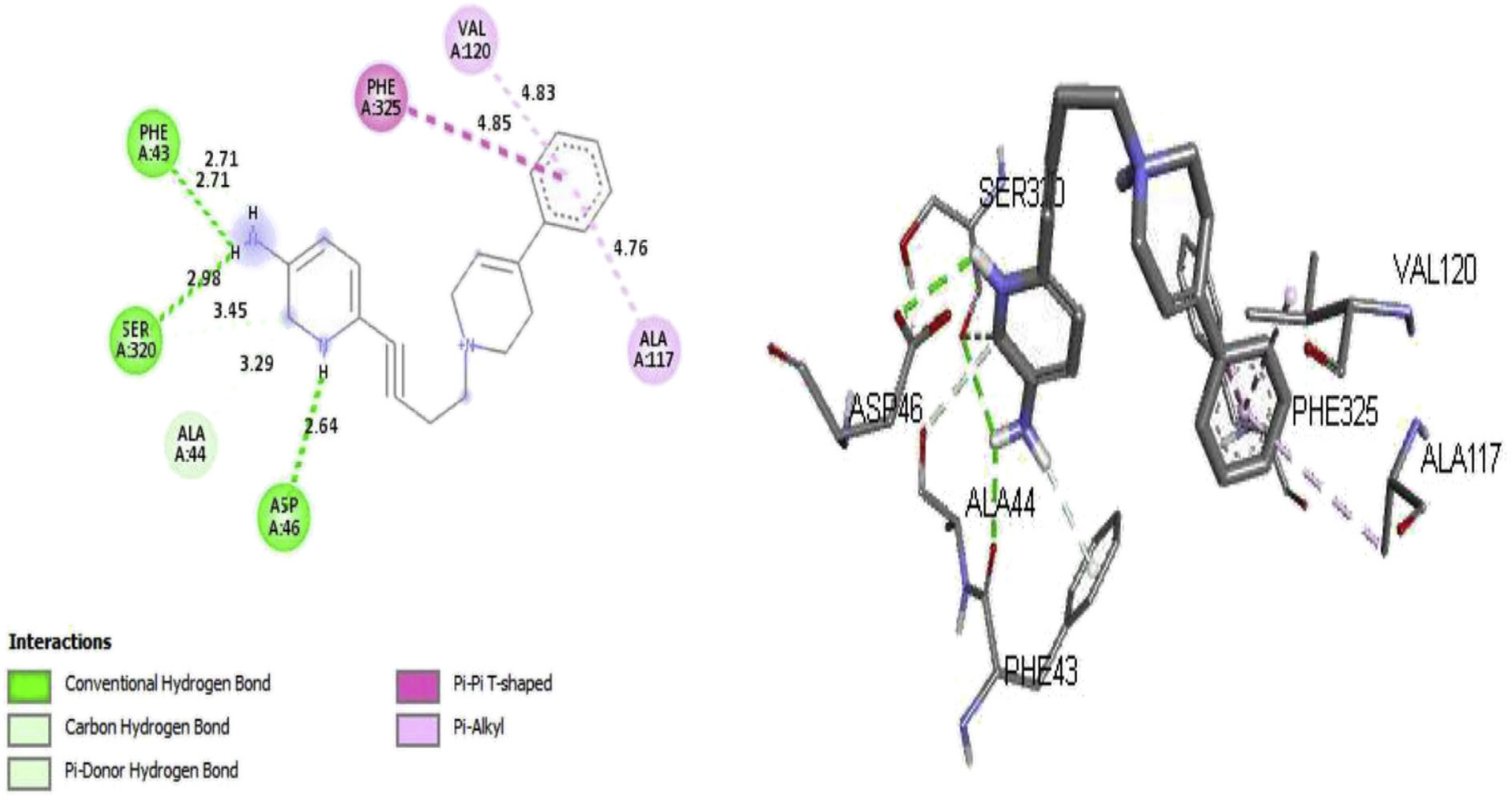
2D and 3D molecular interaction for Complex 28.

**Figure 7:**
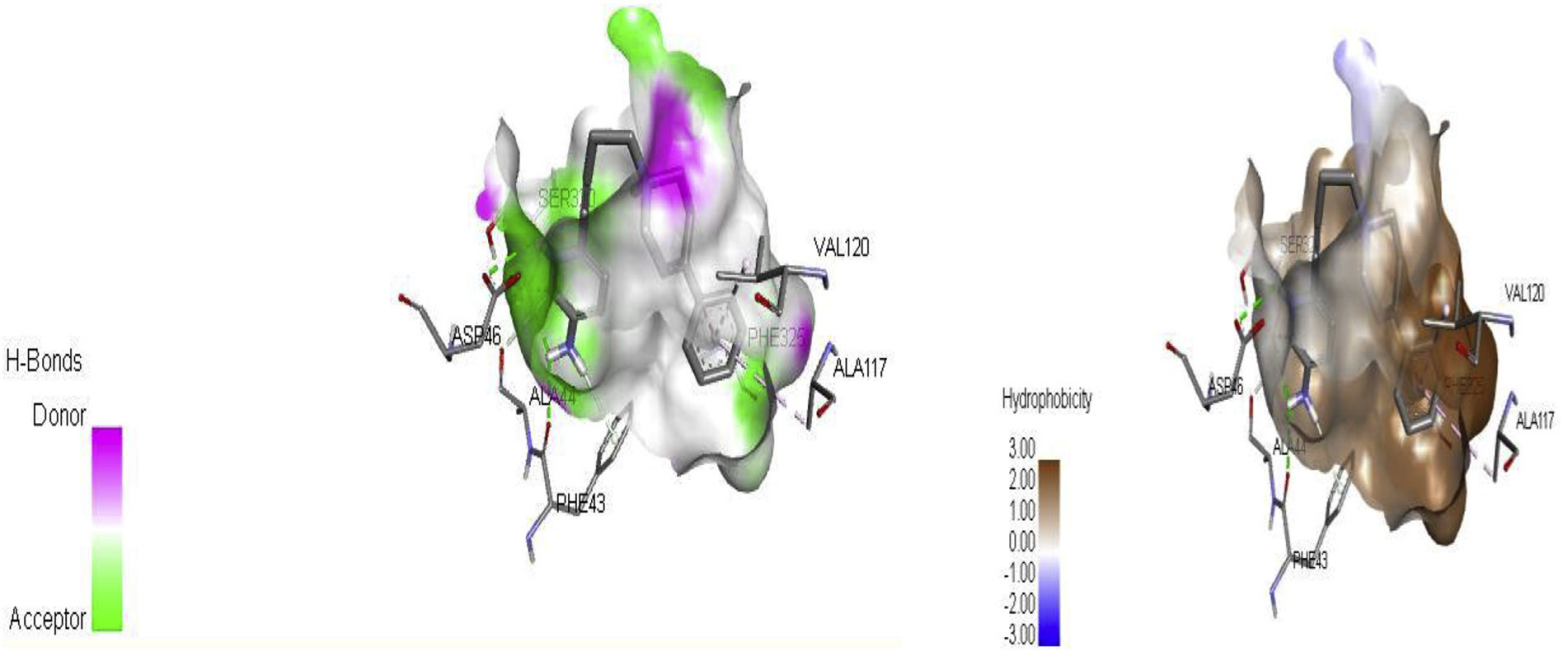
H-bond and Hydrophobic molecular interactions between ligand 28 and the receptor.

Molecular docking analysis was performed on the receptor (protein crystal structure of dopamine transporter elucidates antidepressant mechanism, PDB code 4M48) and the inhibitors (ligands) to elucidate the molecular interactions between the target protein (receptor) and the inhibitors and also to evaluate the performance of Docking Algorithms used in the study. Computed binding affinity (docking scores) of all the complexes which range from -3.9 to -10.05 kcal/mol and their corresponding RMSD values when docking the ligands into the active site of the receptor were contained in Supplementary Table SD1. Some of the selected ligands (1, 21, 28 and 39) of higher binding affinity (-9.0 to -10.05 kcal/mol) and their experimental activity, RMSD values, amino acid residues, bond distance, bond type as well as the type of molecular interactions were presented in Table 6. On visualizing the complexes by using the discovery studio visualizer 2016 version in other to elucidate the type of molecular interactions and binding mode, our findings showed that all the ligands were strongly bounded and fully occupy the active site of the receptor with the formation of major interactions (hydrogen bond, hydrophobic and electrostatic interactions) as reported in Table 6. Ligand 21 with highest docking score (-10.05 kcal/mol) showed two distinct interactions (Hydrogen bond and hydrophobic interactions) with the hydrogen bonding pocket consisting of amino acid residues; TYR123 (2.073 Å), VAL120 (2.073 Å), TRY123 (2.327 Å) and VAL126 (2.327 Å) while the hydrophobic pockets were surrounded by LEU474, ASP475, TYR123 and VAL120 (amino acid residues) of the target protein. More so, all the selected ligands displayed hydrophobic interactions with amino acid residue **VAL120** common to all the selected complexes and the selected complexes have a computed RMSD value ≤ 2Å, this implies a successful and an excellent docking predictions [45]. Figure 6 depicts 2D and 3D molecular interactions of the ligand 28 while Figure 7 shows the hydrogen bond and hydrophobic interactions when the ligand occupied the active site of the target protein (PDB: 4M48). Ligand 28 revealed two significant interactions (Hydrogen bond and hydrophobic) with the formation of the highest number of hydrogen bonds (6 hydrogen bonds) among the selected ligands. The complex (28) formed three (3) Conventional Hydrogen Bonds, two (2) Carbon Hydrogen Bonds and one (1) Pi-Donor Hydrogen Bond surrounded by amino acid residues (ASP46, PHE43, SER320, ALA44, SER320 and PHE43) and with hydrophobic interactions (1 Pi-Pi T-shaped and 2 Pi-Alkyl) surrounded by the amino acid residues (PHE325, ALA117 and VAL120) in the active site of the target protein (Table 6). The observed higher number of hydrogen bonds formed by the complex 28 compare to other selected ligands may inform its higher inhibitory value (pKi = 9.30) of the ligand. The observed hydrogen bond, as well as hydrophobic interactions in the complexes, suggests that the selected ligands are potent inhibitor against the target protein (PDB: 4M48).

**Table 6:** Binding affinity, RMSD values, amino acid residues, bond distance, type of bonds and type of interactions of the selected ligands.

Ligands no	Binding affinity (kcal/mol)	Activity pKi	RMSD value	Amino acids	Bond distance	Type of bond	Type of interaction
21	-10.05	8.05	1.626	TYR123, VAL120	2.07297	Hydrogen Bond	Conventional Hydrogen Bond
				VAL126, TYR123	2.32725	Hydrogen Bond	Conventional Hydrogen Bond
				LEU474; ASP475; TYR123	4.02085	Hydrophobic	Amide-Pi Stacked
				TYR123; VAL120	4.7985	Hydrophobic	Pi-Alkyl
				TYR123 LEU474	5.34773	Hydrophobic	Pi-Alkyl
39	-9.95	9.28	1.749	ARG52	2.17082	Hydrogen Bond	Conventional Hydrogen Bond
				ARG52	2.35234	Hydrogen Bond	Conventional Hydrogen Bond
				PHE319	3.90232	Hydrophobic	Pi-Pi Stacked
				PHE319	3.93778	Hydrophobic	Pi-Pi Stacked
				ALA117	3.40983	Hydrophobic	Alkyl
				ALA428	4.25202	Hydrophobic	Alkyl
				VAL327	4.32679	Hydrophobic	Alkyl
				ALA117	5.1901	Hydrophobic	Pi-Alkyl
				VAL120	4.33937	Hydrophobic	Pi-Alkyl
1	-9.5	7.602	1.229	SER421	2.84844	Hydrogen Bond	Conventional Hydrogen Bond
				ASP46	2.98415	Hydrogen Bond	Conventional Hydrogen Bond
				ASP46	3.72532	Electrostatic	Pi-Anion
				ILE116	3.68729	Hydrophobic	Pi-Sigma
				TYR124	5.0905	Hydrophobic	Pi-Pi Stacked
				PHE325	4.28117	Hydrophobic	Pi-Pi Stacked
				TYR124	4.67821	Hydrophobic	Pi-Pi Stacked
				ALA117	5.47755	Hydrophobic	Pi-Alkyl
				VAL120	4.60622	Hydrophobic	Pi-Alkyl
				ALA479	4.59896	Hydrophobic	Pi-Alkyl
				ILE483	4.94668	Hydrophobic	Pi-Alkyl
28	-9	9.30	1.853	ASP46	2.63847	Hydrogen Bond	Conventional Hydrogen Bond
				PHE43	2.70853	Hydrogen Bond	Conventional Hydrogen Bond
				SER320	2.98218	Hydrogen Bond	Conventional Hydrogen Bond
				ALA44	3.29041	Hydrogen Bond	Carbon Hydrogen Bond
				SER320	3.44795	Hydrogen Bond	Carbon Hydrogen Bond
				PHE43	2.70512	Hydrogen Bond	Pi-Donor Hydrogen Bond
				PHE325	4.84644	Hydrophobic	Pi-Pi T-shaped
				ALA117	4.76375	Hydrophobic	Pi-Alkyl
				VAL120	4.83084	Hydrophobic	Pi-Alkyl

## 4 Conclusion

Chemoinformatic studies were successfully investigated on some inhibitors of Dopamine Transporter (DAT) to develop a predictive, reliable and statistically significant model. The results showed that the model passed the minimum requirements for a globally acceptable QSAR model (R^2^ = 0.802, R^2^adj = 0.767, Q^2^_cv_ = 0.693, R^2^_Test_ = 0.77 and cR^2^p = 0.714). The mechanistic interpretation and elucidation of the descriptors in the model also revealed that two of the molecular descriptors; MATS7s (32.3%) (Moran autocorrelation weighted by van der Waals volumes) and RDF95m (30.4%) (Radial distribution function weighted by relative mass) played predominant roles in the observed anti-psychotic activities of the studied compounds, this implies that in the future design of a novel and highly potent antipsychotic agents, the molecular volume of a compound should be appreciably high while its molecular mass should be significantly low. More so, the predictivity, reliability, stability, robustness, and applicability of the QSAR model were established and the developed model proved to satisfy the OECD principle for a QSAR model development. Furthermore, in elucidating the molecular interactions between the inhibitors and the receptor (PDB:4M48) targeting schizophrenia via Molecular dockings analysis, our findings showed that the study molecules had excellent binding affinities (ranges from-3.9 to -10.05kcl/mol), good docking predictions (RMSD ≤ 2Å) and very significant interactions with the protein target. Likewise, ligand 28 (3-phenoxy-N-(2-(m-tolyloxy)ethyl)propan-1-aminium) with the highest experimental activity (pKi= 9.30) exhibited the highest number of hydrogen bonds when compared to other selected ligands. Hence, the obtained results from this study are envisaged to provide necessary information on the structural requirements and physicochemical parameters/ properties needed to develop novel and more potent antipsychotic therapeutic agents with improved activity for the treatment of schizophrenia and other related mental disorders.

## Declarations

### Author contribution statement

Adamu Uzairu: Conceived and designed the experiments; Contributed reagents, materials, analysis tools or data.

Olasupo Sabitu Babatunde: Performed the experiments; Wrote the paper.

Gedion Adamu Shallawang: Analyzed and interpreted the data; Contributed reagents, materials, analysis tools or data.

Sani Uba: Analyzed and interpreted the data; Wrote the paper.

### Funding statement

This research did not receive any specific grant from funding agencies in the public, commercial, or not-for-profit sectors.

### Competing interest statement

The authors declare no conflict of interest.

### Additional information

No additional information is available for this paper.

## Appendix ASupplementary data

Supplementary Tables SD.doc Supplementary Tables SD.doc
