# Supplementary material for: Chemoinformatic studies on some inhibitors of dopamine transporter and the receptor targeting schizophrenia for developing novel antipsychotic agents
Source: Heliyon. 2020 Jul 28;6(7):e04464. doi: 10.1016/j.heliyon.2020.e04464 (PMC7393552; doi:10.1016/j.heliyon.2020.e04464)
Supplement: Supplementary Tables SD.doc [file mmc1.doc]

**SUPPLEMENTARY TABLES**

**Supplementary Table SD1**

| S/N | ID | IUPAC NAME | STRUCTURE | Experimental Activity pKi | Binding Affinity | Docking RMSD values |
| --- | --- | --- | --- | --- | --- | --- |
|  | CHEMBL59103 | 1-((1H-indol-3-yl)methyl)-4-phenylpiperidin-1-ium |  | 7.602 | -9.5 | 1.229 |
|  | CHEMBL57478 | 1-((1H-indol-3-yl)methyl)-4-methylpiperidin-1-ium |  | 6.244 | -7.9 | 2.145 |
|  | CHEMBL59324 | 1-((1H-indol-3-yl)methyl)-4-phenylpiperazin-1-ium |  | 8.097 | -8.9 | 1.267 |
|  | CHEMBL58296 | 1-(4-chlorobenzyl)-4-(5-(4-chlorophenyl)isoxazol-3-yl)piperidin-1-ium |  | 7.357 | -9 | 1.9 |
|  | CHEMBL60815 | 4-phenyl-1-((4-phenyl-1H-pyrrol-2-yl)methyl)piperazin-1-ium |  | 7.921 | -9.15 | 1.563 |
|  | CHEMBL60518 | 1-((1H-pyrrolo[2,3-b]pyridin-3-yl)methyl)-4-(4-iodophenyl)piperazin-1-ium |  | 9.292 | -8.5 | 1.348 |
|  | CHEMBL61080 | 1-(4-chlorobenzyl)-4-(5-(4-chlorophenyl)-1H-pyrazol-3-yl)piperidin-1-ium |  | 7.215 | -9.7 | 3.848 |
|  | CHEMBL61079 | 4-(5-(4-chlorophenyl)-4-methyl-1H-pyrazol-3-yl)-1-phenethylpiperidin-1-ium |  | 8.921 | -9.6 | 1.827 |
|  | CHEMBL61195 | 1-(4-chlorobenzyl)-4-(3-(4-chlorophenyl)isoxazol-5-yl)piperidin-1-ium |  | 8.444 | -6.6 | 3.843 |
|  | CHEMBL80411 | 1-(4-((2H-benzo[b][1,4]oxazin-3-yl)(methyl)amino)butyl)-4-(pyrimidin-2-yl)piperazin-1-ium |  | 6.253 | -8 | 3.392 |
|  | CHEMBL80655 | 3-((3-(4-(pyrimidin-2-yl)piperazin-1-ium-1-yl)propyl)amino)-2H-benzo[b][1,4]oxazin-4-ium |  | 6.971 | -8.7 | 2.5 |
|  | CHEMBL80645 | 3-((5-(4-(pyrimidin-2-yl)piperazin-1-ium-1-yl)pentyl)amino)-2H-benzo[b][1,4]oxazin-4-ium |  | 7.721 | -8.5 | 2.795 |
|  | CHEMBL80627 | 3-((5-(4-(pyridin-2-yl)piperazin-1-ium-1-yl)pentyl)amino)-2H-benzo[b][1,4]oxazin-4-ium |  | 7.301 | -7.75 | 2.729 |
|  | CHEMBL80711 | 3-((3-(4-(2-methoxyphenyl)piperazin-1-ium-1-yl)propyl)amino)-2H-benzo[b][1,4]oxazin-4-ium |  | 8.523 | -8.65 | 1.672 |
|  | CHEMBL88289 | 1-benzyl-4-(2-(trifluoromethyl)-1H-benzo[d]imidazol-4-yl)piperazin-1-ium |  | 8.538 | -9.3 | 1.849 |
|  | CHEMBL88837 | 4-(1H-benzo[d]imidazol-4-yl)-1-benzylpiperazin-1-ium |  | 7.328 | -8.55 | 2.76 |
|  | CHEMBL90476 | 1-benzyl-4-(7-bromo-1H-indol-4-yl)piperazin-1-ium |  | 8.585 | -9.4 | 1.889 |
|  | CHEMBL99967 | 1-((2-phenyl-1H-imidazol-4-yl)methyl)-4-(pyridin-2-yl)piperazin-1-ium |  | 8.071 | -9 | 3.953 |
|  | CHEMBL103772 | 1-((2-phenyl-1H-imidazol-4-yl)methyl)-4-(pyrimidin-2-yl)piperazin-1-ium |  | 8.420 | -5.9 | 2.252 |
|  | CHEMBL103871 | 4-phenyl-1-((2-phenyl-1H-imidazol-4-yl)methyl)piperazin-1-ium |  | 8.284 | -9.1 | 3.724 |
|  | CHEMBL53 | 10,11-dihydroxy-6-methyl-5,6,6a,7-tetrahydro-4H-dibenzo[de,g]quinolin-6-ium |  | 8.051 | -10.05 | 1.626 |
|  | CHEMBL108463 | 4-phenyl-1-(4-(pyridin-3-yl)but-3-yn-1-yl)-1,2,3,6-tetrahydropyridin-1-ium |  | 6.170 | -8.75 | 1.807 |
|  | CHEMBL108531 | 1-(4-(4-aminophenyl)but-3-yn-1-yl)-4-phenyl-1,2,3,6-tetrahydropyridin-1-ium |  | 6.640 | -9.2 | 0.828 |
|  | CHEMBL109063 | 1-(4-(6-aminopyridin-3-yl)but-3-yn-1-yl)-4-phenyl-1,2,3,6-tetrahydropyridin-1-ium |  | 6.560 | -8.6 | 1.94 |
|  | CHEMBL109101 | 1-(4-(5-aminopyridin-2-yl)but-3-yn-1-yl)-4-phenyl-1,2,3,6-tetrahydropyridin-1-ium |  | 6.430 | -8.9 | 3.636 |
|  | CHEMBL110489 | 4-phenyl-1-(4-(pyridin-4-yl)but-3-yn-1-yl)-1,2,3,6-tetrahydropyridin-1-ium |  | 6.240 | -8.85 | 0.068 |
|  | CHEMBL125916 | 1-(4-(1H-indole-2-carboxamido)butyl)-4-(2-methoxyphenyl)piperazin-1-ium |  | 6.322 | -8.3 | 1.961 |
|  | CHEMBL128232 | 3-phenoxy-N-(2-(m-tolyloxy)ethyl)propan-1-aminium |  | 9.340 | -9 | 1.853 |
|  | CHEMBL128222 | 3-(4-bromophenoxy)-N-(2-(4-chlorophenoxy)ethyl)propan-1-aminium |  | 7.420 | -6.2 | 1.698 |
|  | CHEMBL129534 | N-(2-(3-chlorophenoxy)ethyl)-3-phenoxypropan-1-aminium |  | 9.510 | -6.9 | 2.052 |
|  | CHEMBL128647 | 3-phenoxy-N-(2-(p-tolyloxy)ethyl)propan-1-aminium |  | 9.280 | -4.7 | 8.045 |
|  | CHEMBL128524 | 3-phenoxy-N-(2-phenoxyethyl)propan-1-aminium |  | 8.699 | -3.9 | 4.094 |
|  | CHEMBL130370 | 3-(p-tolyloxy)-N-(2-(p-tolyloxy)ethyl)propan-1-aminium |  | 7.960 | -5.8 | 2.371 |
|  | CHEMBL129757 | 3-(4-chlorophenoxy)-N-(2-(4-chlorophenoxy)ethyl)propan-1-aminium |  | 9.080 | -6.9 | 1.911 |
|  | CHEMBL129927 | N-(2-(3,4-dimethylphenoxy)ethyl)-3-phenoxypropan-1-aminium |  | 9.460 | -7.55 | 1.192 |
|  | CHEMBL131299 | N-(2-phenoxyethyl)-3-(p-tolyloxy)propan-1-aminium |  | 8.570 | -6.35 | 2.368 |
|  | CHEMBL131396 | N-(2-(2-chlorophenoxy)ethyl)-3-phenoxypropan-1-aminium |  | 8.360 | -6.5 | 3.374 |
|  | CHEMBL131484 | N-(2-(4-chlorophenoxy)ethyl)-3-(p-tolyloxy)propan-1-aminium |  | 8.250 | -7.5 | 1.731 |
|  | CHEMBL131997 | N-(2-(3-chloro-4-methylphenoxy)ethyl)-3-(p-tolyloxy)propan-1-aminium |  | 9.280 | -9.95 | 1.749 |
|  | CHEMBL140594 | 4-(4-chlorophenyl)-1-((5-cyano-1H-indol-2-yl)methyl)piperazin-1-ium |  | 8.678 | -8.4 | 1.79 |
|  | CHEMBL140165 | 1-((5-cyano-1H-indol-2-yl)methyl)-4-(4-fluorophenyl)piperazin-1-ium |  | 6.959 | -8.9 | 1.797 |
|  | CHEMBL140258 | 4-(2-chlorophenyl)-1-((6-cyano-1H-indol-2-yl)methyl)piperazin-1-ium |  | 8.469 | -9.1 | 2.491 |
|  | CHEMBL141035 | 4-(2-chlorophenyl)-1-((5-cyano-1H-indol-2-yl)methyl)piperazin-1-ium |  | 8.854 | -7.9 | 1.186 |
|  | CHEMBL243225 | 1-(3-(3-(3,4-dimethoxyphenyl)-4,5-dihydroisoxazol-5-yl)propyl)-4-(2-ethoxyphenyl)piperazin-1-ium |  | 6.648 | -9.65 | 1.862 |

**Supplementary Table SD2: Applicability domain (AD) of model 1 (Training set)**

| Compd | ATS7m | MATS7s | VR2_Dzp | RDF95m | RDF150p | Outlier Info. |
| --- | --- | --- | --- | --- | --- | --- |
| 2 | 0 | 0 | 0 | 0.003 | 0 | within |
| 3 | 0.179 | 0.1 | 0.045 | 0.142 | 0 | within |
| 4 | 0.617 | 0.039 | 0.116 | 0.083 | 0.084 | within |
| 5 | 0.231 | 0.181 | 0.161 | 0.154 | 0.006 | within |
| 6 | 0.836 | 0.07 | 0.055 | 0.068 | 0 | within |
| 7 | 0.622 | 0.048 | 0.11 | 0.063 | 0.054 | within |
| 8 | 0.674 | 0.059 | 0.097 | 0.064 | 0.014 | within |
| 9 | 0.63 | 0.032 | 0.387 | 0.071 | 0.067 | Outlier |
| 10 | 0.44 | 0.096 | 0.092 | 0.109 | 0.251 | within |
| 11 | 0.326 | 0.086 | 0.084 | 0.042 | 0.229 | within |
| 12 | 0.372 | 0.119 | 0.1 | 0.027 | 0.33 | within |
| 13 | 0.37 | 0.122 | 0.093 | 0.055 | 0.224 | within |
| 14 | 0.412 | 0.155 | 0.084 | 0.058 | 0.262 | within |
| 15 | 0.466 | 0.103 | 0.061 | 0.066 | 0 | within |
| 16 | 0.135 | 0.132 | 0.076 | 0.088 | 0 | within |
| 18 | 0.237 | 0.146 | 0.097 | 0.061 | 0 | within |
| 20 | 0.234 | 0.16 | 0.068 | 0.056 | 0 | within |
| 21 | 0.005 | 0.079 | 0.059 | 0 | 0 | within |
| 22 | 0.149 | 0.054 | 0.052 | 0.066 | 0.097 | within |
| 24 | 0.182 | 0.082 | 0.028 | 0.05 | 0.17 | within |
| 25 | 0.185 | 0.094 | 0.027 | 0.076 | 0.217 | within |
| 27 | 0.459 | 0.098 | 0.085 | 0.053 | 0.418 | within |
| 28 | 0.13 | 0.199 | 0.103 | 0.013 | 0.104 | within |
| 29 | 0.383 | 0.172 | 0.045 | 0.113 | 0.091 | within |
| 30 | 0.192 | 0.176 | 0.083 | 0.012 | 0.112 | within |
| 31 | 0.125 | 0.143 | 0.233 | 0.011 | 0.098 | within |
| 32 | 0.09 | 0.171 | 0.042 | 0.009 | 0.092 | within |
| 33 | 0.16 | 0.122 | 0.05 | 0.012 | 0.119 | within |
| 36 | 0.179 | 0.17 | 0.064 | 0.071 | 0.076 | within |
| 38 | 0.214 | 0.142 | 0.045 | 0.01 | 0.117 | within |
| 39 | 0.262 | 0.126 | 0.071 | 0.011 | 0.125 | within |
| 40 | 0.432 | 0.143 | 0.077 | 0.078 | 0.001 | within |
| 41 | 0.347 | 0.054 | 0.067 | 0.074 | 0.019 | within |
| 44 | 0.794 | 0.177 | 0.144 | 0.177 | 0.501 | Outlier |

**Supplementary Table SD3: Applicability domain of model 1 (Test set**)

| Compd | ATS7m | MATS7s | VR2_Dzp | RDF95m | RDF150p | AD information. |
| --- | --- | --- | --- | --- | --- | --- |
| 1 | 0.169 | 0.021 | 0.041 | 0.019 | 0 | within |
| 17 | 0.542 | 0.153 | 0.084 | 0.113 | 0 | within |
| 19 | 0.24 | 0.133 | 0.119 | 0.063 | 0 | within |
| 23 | 0.179 | 0.105 | 0.023 | 0.046 | 0.153 | within |
| 26 | 0.149 | 0.069 | 0.028 | 0.051 | 0.171 | within |
| 34 | 0.268 | 0.165 | 0.045 | 0.01 | 0.091 | within |
| 35 | 0.166 | 0.173 | 0.07 | 0.011 | 0.1 | within |
| 37 | 0.179 | 0.17 | 0.064 | 0.071 | 0.077 | within |
| 42 | 0.337 | 0.147 | 0.134 | 0.144 | 0 | within |
| 43 | 0.337 | 0.147 | 0.099 | 0.137 | 0.043 | within |

**Supplementary Table SD4**: Y-randomization Analysis Parameters

| *Model* | *R* | *R^2* | *Q^2* |
| --- | --- | --- | --- |
| *Original* | 0.895575 | 0.802055 | 0.693313 |
| *Random 1* | 0.389783 | 0.151931 | -0.33728 |
| *Random 2* | 0.360208 | 0.12975 | -0.44387 |
| *Random 3* | 0.187154 | 0.035027 | -0.46452 |
| *Random 4* | 0.515977 | 0.266233 | -0.1147 |
| *Random 5* | 0.430799 | 0.185588 | -0.36844 |
| *Random 6* | 0.328004 | 0.107587 | -0.25517 |
| *Random 7* | 0.490972 | 0.241053 | -0.14152 |
| *Random 8* | 0.445304 | 0.198295 | -0.17069 |
| *Random 9* | 0.459559 | 0.211194 | -0.12819 |
| *Random 10* | 0.478404 | 0.228871 | -0.47775 |
|  |  |  |  |
|  |  |  |  |
| *Random Models Parameters* |  |  |  |
| *Average r :* | 0.408616 |  |  |
| *Average r^2 :* | 0.175553 |  |  |
| *Average Q^2 :* | -0.29021 |  |  |
| *cRp^2 :* | 0.713705 |  |  |

**Supplementary Table SD5: Variance Inflation Factor (VIF) Statistic for the Descriptors in Model 1**

| S/N | Independent variable | R^2^ | VIF |
| --- | --- | --- | --- |
| 1 | ATS7m | 0.4168 | 1.7 |
| 2 | MATS7s | 0.1654 | 1.2 |
| 3 | VR2_Dzp | 0.1292 | 1.1 |
| 4 | RDF95m | 0.2580 | 1.3 |
| 5 | RDF150p | 0.1345 | 1.2 |
